# Lobectomy Versus Sublobectomy in Stage IIIA/N2 Non-Small Cell Lung Cancer: A Population-Based Study

**DOI:** 10.3389/fonc.2021.726811

**Published:** 2021-12-09

**Authors:** Suyu Wang, Zhiyuan Zhang, Yang Gu, Xin Lv, Xuan Shi, Meiyun Liu

**Affiliations:** ^1^ Department of Cardiothoracic Surgery, Changzheng Hospital, Naval Medical University, Shanghai, China; ^2^ Department of Cardiothoracic Surgery, No. 988 Hospital of Joint Logistic Support Force, Zhengzhou, China; ^3^ Department of Anesthesiology, Shanghai Pulmonary Hospital, Tongji University School of Medicine, Shanghai, China

**Keywords:** lobectomy, sublobectomy, IIIA/N2, non-small cell lung cancer, SEER (Surveillance Epidemiology and End Results) database

## Abstract

**Background:**

The role lobectomy plays in stage IIIA/N2 non-small cell lung cancer (NSCLC) is controversial for a long time. What’s more, no previous study concentrates on whether sublobectomy can improve survival outcome for these patients, so we performed this population-based study to investigate whether stage IIIA/N2 NSCLC can benefit from these two surgery types and compare survival outcomes after lobectomy and sublobectomy.

**Methods:**

A total of 21,638 patients diagnosed with stage IIIA/N2 NSCLC between 2004 and 2015 from the Surveillance, Epidemiology, and End Results (SEER) database matched our selection criteria. The study cohort included patients who received no surgery (n = 15,951), sublobectomy (n = 628) and lobectomy (n = 5,059). Kaplan–Meier method, Cox regression analyses, and inverse probability of treatment weighting (IPTW)-adjusted Cox regression were used to illustrate the influence of sublobectomy and lobectomy on overall survival (OS) rates in the study cohort and compare these two surgery types.

**Results:**

Multivariable Cox regression analysis showed sublobectomy [HR: 0.584 (95%CI: 0.531–0.644), P-value <0.001; IPTW-adjusted HR: 0.619 (95%CI: 0.605–0.633), P-value <0.001] and lobectomy [HR: 0.439 (95%CI: 0.420–0.459), P-value <0.001; IPTW-adjusted HR: 0.441 (95%CI: 0.431–0.451), P-value <0.001] were both related to better OS rates compared with no surgery, and lobectomy exhibited better survival than sublobectomy [HR: 0.751 (95%CI: 0.680–0.830), P-value <0.001; IPTW-adjusted HR: 0.713 (95%CI: 0.696–0.731), P-value <0.001]. Moreover, the results in subgroup analyses based on age, tumor size and radiotherapy and chemotherapy strategy in all study cohort were consistent.

**Conclusion:**

Stage IIIA/N2 NSCLC patients could benefit from sublobectomy or lobectomy, and lobectomy provided better OS rates than sublobectomy.

## Introduction

Lung cancer is one of the most common malignancies with the highest morbidity and mortality around the world. Non-small cell lung cancer (NSCLC) is the most frequent subtype, which accounts for approximately 80–85% of lung cancer (1), and 10–20% of NSCLC patients are diagnosed with N2-positve clinical stage IIIA (IIIA/N2) disease (2). The treatment modalities for stages I, II, IIIB, and IV NSCLC have been sufficiently studied and applied worldwide. However, therapeutic strategies for IIIA/N2 NSCLC are still controversial and differ among centers, countries, and continents (3). Several studies have suggested concurrent chemoradiotherapy or surgery combing chemotherapy or chemoradiotherapy (4, 5). Chemotherapy is the optimal method for potentially distant metastasis, and radiotherapy or surgery can be combined or chosen singly to treat local tumor. Surgery is the most effective strategy for radically treating cancer if a complete dissection can be safely achieved. However, the role surgery plays in IIIA/N2 NSCLC has been debated for decades and no consensus has been reached.

Although the National Comprehensive Cancer Network (NCCN) guidelines for NSCLC provides no clear recommendation for surgery on IIIA/N2 NSCLC, 90.5% of the institutions belonging to the NCCN would consider surgery in patients with only one N2 lymph node station smaller than 3 cm involved, while 47.6% of the institutions consider surgery in multi-station involved patients with lymph nodes smaller than 3 cm, and 16.7% of the institutions still consider surgery for multi-station involved patients with lymph nodes larger than 3 cm (6, 7). Regarding surgery on IIIA/N2 NSCLC, two classic randomized clinical trials have to be mentioned. Van Meerbeeck’s team published the largest bimodality trial which compared the prognosis of radiotherapy with surgery in patients diagnosed at unresectable stage IIIA/N2 who presented a response to induction chemotherapy, demonstrating surgery could not improve progression-free survival or overall survival (OS) (8). Another trial published by Albain’s team studied chemotherapy combining radiotherapy with or without surgery in potentially technically resectable IIIA/N2 NSCLC, revealing the progression-free survival was better in the surgery group than the non-surgery group. However, the two groups showed no significant difference in OS. In subgroup analysis, patients who underwent lobectomy had better OS compared to chemotherapy combining radiotherapy, while OS was not improved by pneumonectomy (9). As this population is heterogeneous, the panel at the NCCN believes that these trials did not sufficiently evaluate the nuances present with the heterogeneity of N2 disease and the potential oncologic benefit of surgery in specific clinical situations. Because of the difficulties to start and implement a randomized controlled trial for IIIA/N2 disease, some researchers come to dig the clinical records of centers or population-based database. Taylor et al. studied 107 patients at The University of Texas M. D. Anderson Cancer Center, and demonstrated patients who underwent induction chemotherapy and surgery (n=55) or concurrent chemoradiotherapy (n = 52) had no significant differences in local control and median overall, 5-year overall, distant metastasis-free, and disease-free survival (10). Another retrospective single-center study included 53 patients with stage IIIA/N2 NSCLC. Nineteen of the patients in group 1 underwent induction chemotherapy and surgery, 14 in group 2 underwent sequential chemoradiotherapy, and 20 in group 3 underwent concurrent chemoradiotherapy. Groups 1 and 3 performed better than group 2, while groups 1 and 3 had similar prognosis (11). While several other retrospective researches recommended surgery after induction chemotherapy/chemoradiotherapy for this population as long as pneumonectomy not performed (12–17). All these studies focused on lobectomy or pneumonectomy and presented inconsistent results, so we performed this research in a population-based cohort to investigate whether stage IIIA/N2 NSCLC patients could benefit from sublobectomy or lobectomy and compare the outcomes of sublobectomy and lobectomy.

## Material and Methods

### Data Source

Data were collected from the Surveillance, Epidemiology, and End Results (SEER) database which included tumor patient data from 18 cancer registries of the National Cancer Institute. We used the SEER*Stat software (version 8.3.9; https://seer.cancer.gov/resources/) to extract information of stage IIIA/N2 [T1-3N2M0 based on the American Joint Committee on Cancer (AJCC) 7th TNM staging system] NSCLC patients diagnosed at 2004–2015 (user name: 14993-Nov2019). Histologic type and site of tumor were coded by the 3rd edition of the International Classification of Diseases for Oncology (ICD-O-3). All SEER data were deidentified before release and contained no personally identifying information of patients, so the requirement for informed consent of the patients and approval of the institutional review committee was waived. This study was implemented in accordance with the Declaration of Helsinki (as revised in 2013) and the Harmonized Tripartite Guideline for Good Clinical Practice from the International Conference on Harmonization.

### Cohort Selection

Samples meeting these following criteria included: (I) patients diagnosed with stage IIIA/N2 (T1-3N2M0 based on the AJCC 7th TNM staging system) primary lung tumor between 2004 and 2015 with site codes as C34.0–C34.9; (II) with histologic type as adenocarcinoma (8015, 8050, 8140, 8141, 8143–8145, 8147, 8190, 8201, 8211, 8250–8255, 8260, 8290, 8310, 8320, 8323, 8333, 8401, 8440, 8470, 8471, 8480, 8481, 8490, 8503, 8507, 8550, 8570–8572, 8574, and 8576), squamous cell carcinoma (8051, 8052, 8070-8076, 8078, 8083, 8084, 8090, 8094, and 8123), and other NSCLC (8003, 8004, 8012–8014, 8021, 8022, 8030–8035, 8046, 8082, 8120, 8200, 8240, 8241, 8243–8246, 8249, 8430, 8525, 8560, 8562, and 8575) based on ICD-O-3 His/Behave, malignant (18); (III) identified as only one primary tumor; (IV) aged ≥18 years; and (V) treated with no surgery, sublobectomy (wedge resection and segmentectomy) or lobectomy. The exclusion criteria were: (I) patients diagnosed with autopsy/death certificate only or with no positive pathologic confirmation; (II) had survival time of less than 1 month after diagnosis; and (III) had missing information about variables required by our study.

### Study Variables and Outcome

The following information was extracted: patient ID, year of diagnosis, age at diagnosis, gender, race, marital status at diagnosis, laterality, primary site, histologic type, differentiation, T classification, tumor size, regional nodes examined, regional nodes positive, radiotherapy, chemotherapy, surgery, survival month, and vital status. The terms “CS Tumor Size/Ext Eval” and “CS Lymph Nodes Eval” were used to maximally identify the patients who underwent neoadjuvant therapy or not for sensitivity analysis. What’s more, age at diagnosis, regional nodes examined, and regional nodes positive was trichotomized using the cohort underwent surgery *via* the X-tile software (version 3.6.1; https://medicine.yale.edu/lab/rimm/research/software/) based on the maximal log-rank χ^2^ value, representing the greatest group difference in survival prognosis (19). Tumor size was divided into four groups according to the previous study (20).

In our research, OS was chosen as the endpoint. OS was defined as the survival months from diagnosis to all-cause death. The survival information of patients from the SEER database is updated annually and the latest follow-up data was released in December 31, 2016.

### Statistical Analysis

To simplify the analysis, all the continuous variables were transformed into categorical variables and displayed as counts and percentages. Baseline data of patients stratified by surgery strategy were compared by using a Pearson’s χ^2^ test or a Fisher’s exact test. Survival curves were presented using the Kaplan–Meier method, and compared by a two-sided log-rank test. Multivariable Cox regression analysis was also used in subgroup analyses. All variables were put into univariable Cox regression analysis. Variables were included as potential confounders in the multivariable Cox regression analysis if they changed effect estimates of surgery on OS by more than 10% or were significantly associated with OS with P-value <0.1 (21). Regional nodes examined and regional nodes positive were not included as confounders to adjust the effect of surgery for these two variables were closely related to the surgery strategy (22). To explore the impact of regional nodes examined or regional nodes positive on OS, they’re put into multivariable Cox analysis separately, and the potential confounders were selected using the similar criteria for surgery strategy. Subgroups analyses were performed using multivariable Cox regression analysis. To better balance the baseline of patients, propensity scores were calculated using generalized boosted models, and inverse probability of treatment weighting (IPTW) was used to adjust the Cox regression analyses (23, 24). Absolute standardized difference was calculated for balance measurement of baseline characteristics before and after IPTW, and values below 0.1 indicated good balance.

All statistical analyses were performed with R software (version 4.0.4; http://www.r-project.org) and EmpowerStats (version 2.0; http://www.empowerstats.com). All tests were two-sided and statistically significant was defined as P-value <0.05.

## Results

### Patient Characteristics

Between January 2004 and December 2015, the SEER database collected 38,639 patients diagnosed with stage IIIA/N2 NSCLC. As shown in **Table S1**, after employing the selection flow, 21,638 patients were included in our study cohort. A total of 15,951 patients had no surgery, 628 underwent sublobectomy (517 with wedge resection and 111 with segmentectomy) and 5,059 received lobectomy. Also, 13,692 patients of the no surgery group, 458 of the sublobectomy group, and 3,235 of the lobectomy group died before the final follow-up. The median follow-up time was 81 months and the median OS was 17 months. As we can see in **Table 1**, patients who underwent surgery were more likely to be younger, had adenocarcinoma, had lower T classification, had smaller tumor size and had more regional nodes examined. Comparing with sublobectomy, patients who underwent lobectomy has the tendency to have higher T classification, larger tumor size and more regional nodes examined.

**Table 1:** Baseline characteristics of IIIAN2 stage NSCLC patients.

Variables	Non-surgery	Sublobectomy	Lobectomy	P
	n = 15,951	n = 628	n = 5,059	
Year of diagnosis				<0.001
2004–2009	7,543 (47.3)	337 (53.7)	2,543 (50.3)	
2010–2015	8,408 (52.7)	291 (46.3)	2,516 (49.7)	
Age				<0.001
<65 years old	5,199 (32.6)	239 (38.1)	2,269 (44.9)	
66–72 years old	4,359 (27.3)	176 (28.0)	1,534 (30.3)	
>72 years old	6,393 (40.1)	213 (33.9)	1,256 (24.8)	
Gender				<0.001
Male	8,886 (55.7)	309 (49.2)	2,440 (48.2)	
Female	7,065 (44.3)	319 (50.8)	2,619 (51.8)	
Race				<0.001
White	12,785 (80.2)	521 (83.0)	4,141 (81.9)	
Black	2,175 (13.6)	70 (11.1)	474 (9.4)	
Other	991 (6.2)	37 (5.9)	444 (8.8)	
Marital status				<0.001
Married	8,298 (52.0)	351 (55.9)	3,189 (63.0)	
Unmarried	2,153 (13.5)	78 (12.4)	545 (10.8)	
Seperated/Divorced/Widowed	5,500 (34.5)	199 (31.7)	1,325 (26.2)	
Laterality				<0.001
Right	10,258 (64.3)	312 (49.7)	3,006 (59.4)	
Left	5,693 (35.7)	316 (50.3)	2,053 (40.6)	
Primary site				<0.001
Main bronchus	663 (4.2)	1 (0.2)	14 (0.3)	
Upper lobe	9,803 (61.5)	422 (67.2)	2,969 (58.7)	
Middle lobe	707 (4.4)	25 (4.0)	263 (5.2)	
Lower lobe	4,286 (26.9)	166 (26.4)	1,707 (33.7)	
Overlapping lesion of lung	118 (0.7)	6 (1.0)	70 (1.4)	
Unknown	374 (2.3)	8 (1.3)	36 (0.7)	
Histologic type				<0.001
Adenocarcinoma	5,825 (36.5)	398 (63.4)	3,302 (65.3)	
Squamous cell	6,254 (39.2)	121 (19.3)	1,007 (19.9)	
Other	3,872 (24.3)	109 (17.4)	750 (14.8)	
Differentiation				<0.001
Grade I	424 (2.7)	41 (6.5)	256 (5.1)	
Grade II	2,457 (15.4)	208 (33.1)	1,930 (38.1)	
Grade III	4,952 (31.0)	302 (48.1)	2,189 (43.3)	
Grade IV	273 (1.7)	10 (1.6)	116 (2.3)	
Unknown	7,845 (49.2)	67 (10.7)	568 (11.2)	
T				<0.001
T1	3,306 (20.7)	262 (41.7)	1,400 (27.7)	
T2	7,401 (46.4)	250 (39.8)	2,653 (52.4)	
T3	5,244 (32.9)	116 (18.5)	1,006 (19.9)	
Tumor size				<0.001
≤1 cm	246 (1.5)	59 (9.4)	81 (1.6)	
>1 cm, ≤ 2cm	1,722 (10.8)	227 (36.1)	878 (17.4)	
>2 cm, ≤ 3cm	2,652 (16.6)	182 (29.0)	1,288 (25.5)	
>3 cm	11,331 (71.0)	160 (25.5)	2,812 (55.6)	
Regional nodes examined				<0.001
≤5	12,935 (81.1)	406 (64.6)	1,012 (20.0)	
6–11	224 (1.4)	95 (15.1)	1,675 (33.1)	
≥12	133 (0.8)	55 (8.8)	1,872 (37.0)	
Unknown	2,659 (16.7)	72 (11.5)	500 (9.9)	
Regional nodes positive				<0.001
≤2	1791 (11.2)	359 (57.2)	2476 (48.9)	
3–4	218 (1.4)	60 (9.6)	1017 (20.1)	
≥5	114 (0.7)	38 (6.1)	1099 (21.7)	
Unknown	13,828 (86.7)	171 (27.2)	467 (9.2)	
Radiotherapy or chemotherapy				<0.001
No	3,003 (18.8)	176 (28.0)	1,123 (22.2)	
Radiotherapy	2,161 (13.5)	37 (5.9)	169 (3.3)	
Chemotherapy	1,900 (11.9)	148 (23.6)	1,546 (30.6)	
Both	8,887 (55.7)	267 (42.5)	2,221 (43.9)	

Categorical variables are presented with number (percentage). NSCLC, non-small cell lung cancer.

### Univariable and Multivariable Analyses

**Figure 1:**
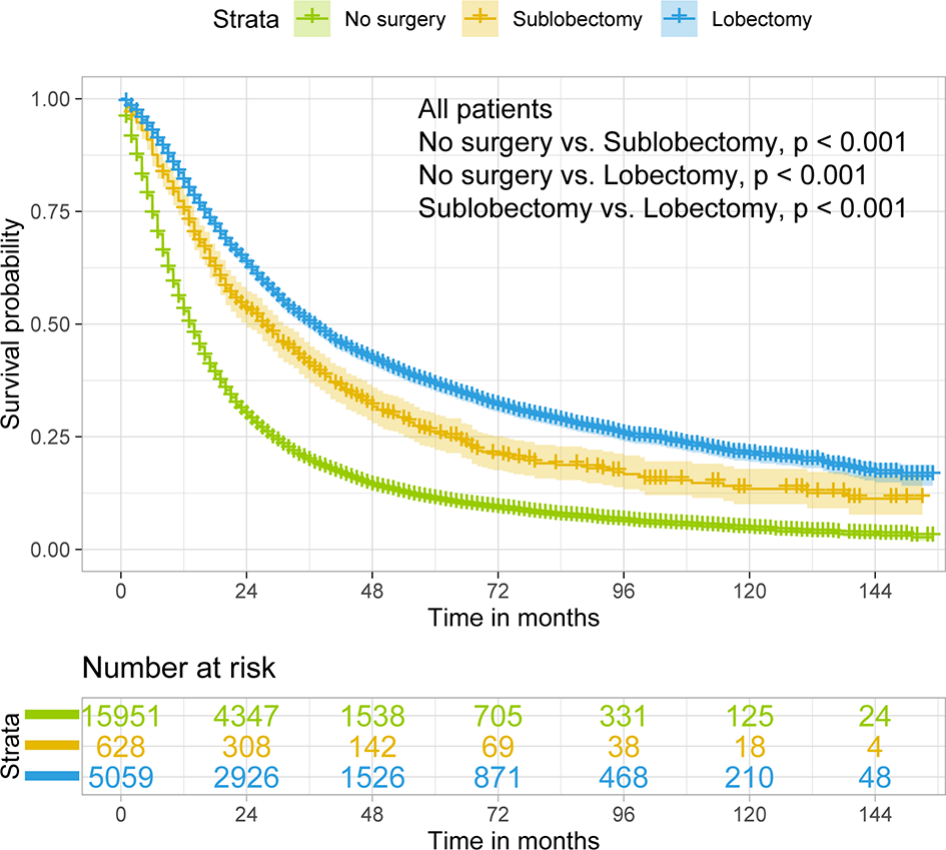
Kaplan–Meier estimates of OS for stage IIIA/N2 NSCLC patients stratified by surgery strategy. OS, overall survival; NSCLC, non-small cell lung cancer.

Both sublobectomy and lobectomy had a better OS compared with no surgery, and lobectomy had improved prognosis compared with sublobectomy (**Figure 1**, all pairwise P-value <0.001). The median survival of patients with no surgery, sublobectomy, and lobectomy was 14, 28, and 37 months respectively. The 5-year OS rates [95% confidence interval (95%CI)] in these three groups were 11.0% (10.4–11.6%), 25.7% (22.1–29.8%), and 36.4% (34.9–37.9%) respectively. The balance measurement of IPTW for analysis of surgical strategy, regional nodes examined and regional nodes positive showed most absolute standardized difference values were below 0.1 after IPTW (**Figure S1**). The univariable analysis in all study cohort revealed patients who underwent surgery, with later year of diagnosis, smaller age, female gender, non-white race, marital status other than separated/divorced/widowed, left side disease, primary site other than main bronchus, adenocarcinoma, lower grade of differentiation, lower T classification, smaller tumor size, more regional nodes examined, less regional nodes positive, chemotherapy or chemoradiotherapy were significantly related with higher OS rates (**Table 2**). As shown in **Tables 2**, **3**, after adjustment by confounders, the hazard ratios (HR) of sublobectomy and lobectomy compared with no surgery was 0.584 (95%CI: 0.531–0.644, P-value <0.001) and 0.439 (95%CI: 0.420–0.459, P-value <0.001) before IPTW and 0.619 (95%CI: 0.605–0.633, P-value <0.001) and 0.441 (95%CI: 0.431–0.451, P-value <0.001) after IPTW respectively. The adjusted HR (95%CI) for lobectomy compared with sublobectomy was 0.754 (95%CI: 0.681–0.836, P-value <0.001) before IPTW and 0.713 (95%CI: 0.696–0.731, P-value <0.001) after IPTW (**Tables 2**, **3**). The impact of regional nodes examined or regional nodes positive on survival of surgery cohort is shown in **Tables S2**–**S5**, and the multivariable Cox regression analyses indicated HRs for regional nodes examined 6–11 and ≥12 compared with ≤5 were 0.877 (95%CI: 0.805–0.955, P-value = 0.003) and 0.784 (95%CI: 0.720–0.855, P-value <0.001) before IPTW and 0.873 (95%CI: 0.834–0.914, P-value <0.001) and 0.780 (95%CI: 0.745–0.817, P-value <0.001) after IPTW. And the HRs for regional nodes positive 3–4 and ≥5 compared with ≤2 were 1.193 (95%CI: 1.093–1.304, P-value <0.001) and 1.509 (95%CI: 1.386–1.643, P-value <0.001) before IPTW and 1.185 (95%CI: 1.130–1.242, P-value <0.001) and 1.490 (95%CI: 1.422–1.561, P-value <0.001) after IPTW. Notably, for patients underwent surgery, single radiotherapy did not confer survival benefit with all HR close to 1 in **Tables S2–** **S5**.

**Table 2:** Cox regression analysis of all IIIAN2 stage NSCLC patients before IPTW.

Variables	Univariable analysis		Multivariable analysis	
	HR (95%CI)	P	HR (95%CI)	P
Surgery				
Sublobectomy vs. no surgery	0.563 (0.513–0.618)	<0.001	0.584 (0.531–0.644)	<0.001
Lobectomy vs. no surgery	0.430 (0.414–0.447)	<0.001	0.439 (0.420–0.459)	<0.001
Lobectomy vs. sublobectomy	0.764 (0.693–0.842)	<0.001	0.751 (0.680–0.830)	<0.001
Year of diagnosis				
2004–2009	1		1	
2010–2015	0.888 (0.862–0.916)	<0.001	0.898 (0.870–0.926)	<0.001
Age				
<65 years old	1		1	
66–72 years old	1.261 (1.213–1.310)	<0.001	1.177 (1.132–1.224)	<0.001
>72 years old	1.711 (1.651–1.772)	<0.001	1.359 (1.308–1.411)	<0.001
Gender				
Male	1		1	
Female	0.801 (0.777–0.825)	<0.001	0.831 (0.805–0.858)	<0.001
Race				
White	1		1	
Black	0.951 (0.909–0.995)	0.030	0.929 (0.887–0.974)	0.002
Other	0.817 (0.769–0.869)	<0.001	0.852 (0.801–0.906)	<0.001
Marital status				
Married	1		1	
Unmarried	1.016 (0.970–1.065)	0.505	1.032 (0.984–1.084)	0.197
Seperated/Divorced/Widowed	1.194 (1.156–1.233)	<0.001	1.112 (1.074–1.151)	<0.001
Laterality				
Right	1		1	
Left	0.969 (0.940–0.999)	0.045	1.000 (0.969–1.031)	0.983
Primary site				
Main bronchus	1		1	
Upper lobe	0.787 (0.724–0.856)	<0.001	0.951 (0.874–1.035)	0.249
Middle lobe	0.786 (0.706–0.875)	<0.001	1.019 (0.913–1.136)	0.739
Lower lobe	0.860 (0.789–0.938)	<0.001	1.062 (0.973–1.159)	0.175
Overlapping lesion of lung	0.817 (0.684–0.976)	0.026	1.026 (0.859–1.226)	0.774
Unknown	1.065 (0.934–1.216)	0.347	1.086 (0.951–1.240)	0.222
Histologic type				
Adenocarcinoma	1		1	
Squamous cell	1.510 (1.460–1.563)	<0.001	1.144 (1.104-1.186)	<0.001
Other	1.375 (1.323–1.429)	<0.001	1.096 (1.052–1.142)	<0.001
Differentiation				
Grade I	1		1	
Grade II	1.161 (1.058–1.273)	0.002	1.243 (1.132–1.365)	<0.001
Grade III	1.368 (1.250–1.498)	<0.001	1.325 (1.209–1.451)	<0.001
Grade IV	1.523 (1.327–1.748)	<0.001	1.464 (1.273–1.684)	<0.001
Unknown	1.522 (1.391–1.665)	<0.001	1.214 (1.108–1.330)	<0.001
T				
T1	1		1	
T2	1.295 (1.246–1.346)	<0.001	1.083 (1.022–1.147)	0.007
T3	1.587 (1.522–1.656)	<0.001	1.278 (1.205–1.355)	<0.001
Tumor size				
≤1 cm	1		1	
>1 cm, ≤2 cm	1.004 (0.883–1.142)	0.948	0.998 (0.878–1.135)	0.977
>2 cm, ≤3 cm	1.225 (1.081–1.389)	0.002	1.167 (1.029–1.325)	0.016
>3 cm	1.589 (1.407–1.796)	<0.001	1.284 (1.128–1.462)	<0.001
Radiotherapy or chemotherapy				
No	1		1	
Radiotherapy	0.481 (0.454–0.509)	<0.001	0.769 (0.729–0.812)	<0.001
Chemotherapy	0.437 (0.412–0.463)	<0.001	0.660 (0.628–0.694)	<0.001
Both	0.735 (0.703–0.768)	<0.001	0.508 (0.488–0.529)	<0.001

NSCLC, non-small cell lung cancer; IPTW, inverse probability of treatment weighting; HR, hazard ratio; CI, confidence interval.

**Table 3:** Cox regression analysis of all IIIAN2 stage NSCLC patients after IPTW.

Variables	Univariable analysis		Multivariable analysis	
	HR (95%CI)	P	HR (95%CI)	P
Surgery				
Sublobectomy vs. no surgery	0.629 (0.615–0.643)	<0.001	0.619 (0.605–0.633)	<0.001
Lobectomy vs. no surgery	0.455 (0.445–0.465)	<0.001	0.441 (0.431–0.451)	<0.001
Lobectomy vs. sublobectomy	0.724 (0.706–0.741)	<0.001	0.713 (0.696–0.731)	<0.001
Year of diagnosis				
2004–2009	1		1	
2010–2015	0.843 (0.827–0.859)	<0.001	0.842 (0.826–0.859)	<0.001
Age				
<65 years old	1		1	
66–72 years old	1.234 (1.205–1.263)	<0.001	1.226 (1.196–1.256)	<0.001
>72 years old	1.628 (1.593–1.665)	<0.001	1.440 (1.405–1.475)	<0.001
Gender				
Male	1		1	
Female	0.859 (0.843–0.875)	<0.001	0.847 (0.830–0.865)	<0.001
Race				
White	1		1	
Black	0.957 (0.929–0.985)	0.003	1.013 (0.982–1.044)	0.420
Other	0.695 (0.667–0.724)	<0.001	0.678 (0.651–0.707)	<0.001
Marital status				
Married	1		1	
Unmarried	0.872 (0.846–0.899)	<0.001	0.928 (0.900–0.957)	<0.001
Seperated/Divorced/Widowed	1.136 (1.113–1.159)	<0.001	1.103 (1.079–1.128)	<0.001
Laterality				
Right	1		1	
Left	0.949 (0.931–0.968)	<0.001	0.984 (0.965–1.004)	0.116
Primary site				
Main bronchus	1		1	
Upper lobe	0.977 (0.928–1.028)	0.372	1.152 (1.089–1.219)	<0.001
Middle lobe	0.978 (0.916–1.043)	0.494	1.200 (1.119–1.287)	<0.001
Lower lobe	1.124 (1.066–1.185)	<0.001	1.302 (1.227–1.381)	<0.001
Overlapping lesion of lung	1.195 (1.059–1.349)	0.004	1.358 (1.200–1.536)	<0.001
Unknown	1.281 (1.167–1.407)	<0.001	1.370 (1.244–1.510)	<0.001
Histologic type				
Adenocarcinoma	1		1	
Squamous cell	1.232 (1.206–1.258)	<0.001	1.094 (1.069–1.118)	<0.001
Other	1.084 (1.058–1.110)	<0.001	1.016 (0.990–1.042)	0.233
Differentiation				
Grade I	1		1	
Grade II	1.263 (1.191–1.339)	<0.001	1.176 (1.109–1.248)	<0.001
Grade III	1.431 (1.351–1.515)	<0.001	1.341 (1.265–1.421)	<0.001
Grade IV	1.643 (1.504–1.796)	<0.001	1.579 (1.442–1.728)	<0.001
Unknown	1.193 (1.127–1.264)	<0.001	1.122 (1.059–1.190)	<0.001
T				
T1	1		1	
T2	1.388 (1.355–1.423)	<0.001	1.215 (1.173–1.259)	<0.001
T3	1.585 (1.544–1.628)	<0.001	1.482 (1.430–1.537)	<0.001
Tumor size				
≤1 cm	1		1	
>1 cm, ≤2 cm	0.942 (0.867–1.023)	0.154	0.894 (0.823–0.971)	0.008
>2 cm, ≤3 cm	1.130 (1.041–1.226)	0.003	1.043 (0.961–1.132)	0.313
>3 cm	1.405 (1.298–1.521)	<0.001	1.119 (1.029–1.216)	<0.001
Radiotherapy or chemotherapy				
No	1		1	
Radiotherapy	1.079 (1.043–1.116)	<0.001	0.961 (0.928–0.995)	0.025
Chemotherapy	0.613 (0.595–0.632)	<0.001	0.635 (0.615–0.654)	<0.001
Both	0.622 (0.607–0.637)	<0.001	0.618 (0.602–0.634)	<0.001

NSCLC, non-small cell lung cancer; IPTW, inverse probability of treatment weighting; HR, hazard ratio; CI, confidence interval.

### Subgroup and Sensitivity Analysis

**Figure 2:**
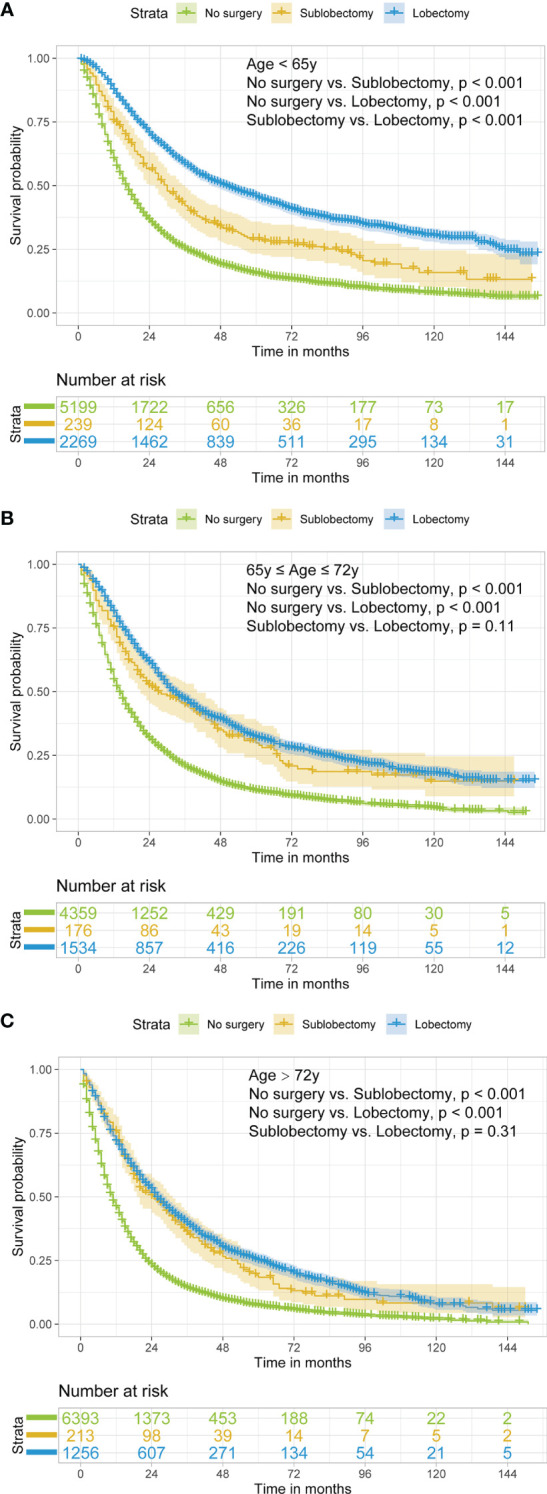
Kaplan–Meier estimates of OS for stage IIIA/N2 NSCLC patients aged <65 years old **(A)**, 65–72 years old **(B)**, and >72 years old **(C)** stratified by surgery strategy. OS, overall survival; NSCLC, non-small cell lung cancer.

**Figure 3:**
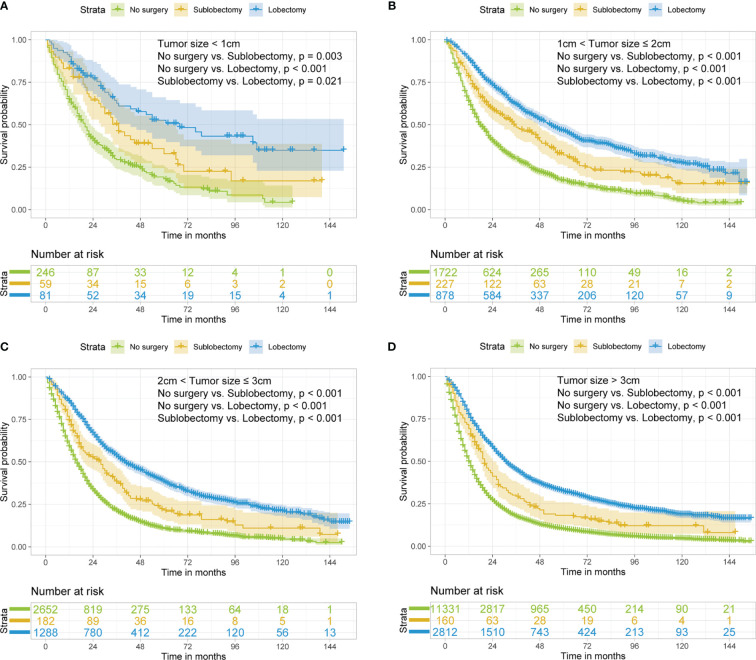
Kaplan–Meier estimates of OS for stage IIIA/N2 NSCLC patients with tumor size <1 cm **(A)**, 1 cm < tumor size ≤2 cm **(B)**, 2 cm < tumor size ≤3 cm **(C)**, tumor size >3 cm **(D)** stratified by surgery strategy. OS, overall survival; NSCLC, non-small cell lung cancer.

**Figure 4:**
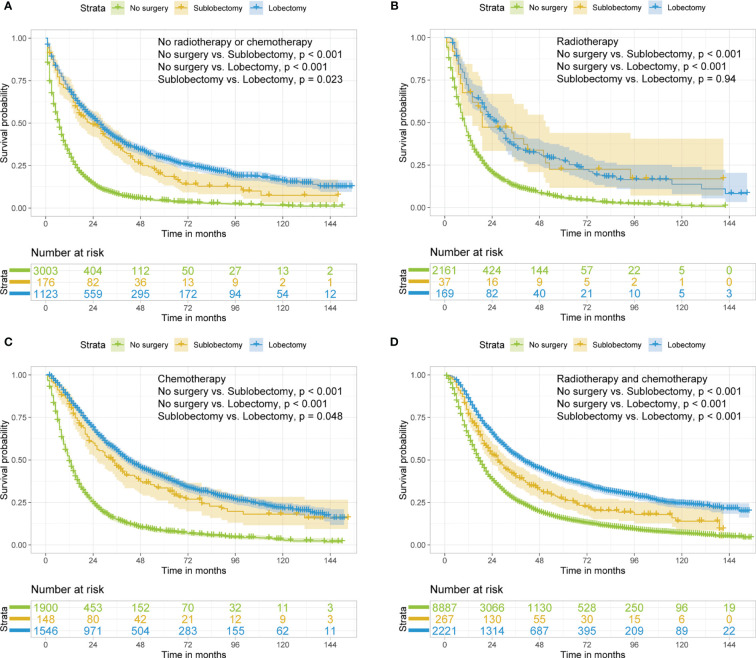
Kaplan–Meier estimates of OS for stage IIIA/N2 NSCLC patients with no radiotherapy or chemotherapy **(A)**, radiotherapy **(B)**, chemotherapy **(C)**, radiotherapy and chemotherapy **(D)** stratified by surgery strategy. OS, overall survival; NSCLC, non-small cell lung cancer.

In subgroup analyses, the surgery groups demonstrated better OS than no surgery group in all age, tumor size, and radiotherapy or chemotherapy subgroups (**Figures 2** **–** **4**, all pairwise P-value <0.005). After adjustment by confounders, the OS of sublobectomy and lobectomy groups was still superior to no surgery group in all subgroups before and after IPTW (**Tables 4**, **5**, all pairwise P-value <0.01). While comparing two surgery types, for patients aged <65 years, the sublobectomy group had worse prognosis than lobectomy group (**Figure 2A**, P-value <0.001), but the difference between the two surgery strategies’ OS were not statistically significant in patients aged 65–72 years (**Figure 2B**, P-value = 0.11) and aged >72 years (**Figure 2C**, P-value = 0.31). The sublobectomy group also had worse prognosis in all tumor size subgroups (**Figure 3**, all pairwise P-value <0.05). In radiotherapy or chemotherapy subgroup analyses, lobectomy achieved better OS than sublobectomy in patients who had no radiotherapy or chemotherapy, and the similar results could be seen in patients who had single chemotherapy or radiotherapy plus chemotherapy (**Figures 4A, C, D**, all pairwise P-value <0.05). The outcomes of sublobectomy and lobectomy were comparable for patients underwent single radiotherapy, however, this result was underpowered for limited samples (**Figure 4B**, all pairwise P-value = 0.94). After adjustment by confounders, as shown in **Tables 4**, **5**, lobectomy still showed superior survival outcome to sublobectomy in all subgroups with all HR <1 and most P-values <0.001. In the sensitivity analysis, comparing to patients who underwent no surgery, patients who underwent surgery with or without neoadjuvant therapy both had HR <1 with P-values <0.005 (**Tables S6**, **S7**). In patients who had surgery with or without neoadjuvant therapy, comparing to patients who underwent sublobectomy, these had lobectomy showed better survival outcome with HR <1, despite the P-value for HR comparing lobectomy with sublobectomy in patients with neoadjuvant therapy was 0.126 (**Tables S6**, **S7**).

**Table 4:** Multivariable Cox regression analysis of subgroups of all IIIAN2 stage NSCLC patients before IPTW.

Subgroups	Sublobectomy vs. no surgery		Lobectomy vs. no surgery		Lobectomy vs. sublobectomy	
	HR (95%CI)	P	HR (95%CI)	P	HR (95%CI)	P
Age						
<65 years old	0.667 (0.568–0.783)	<0.001	0.408 (0.380–0.439)	<0.001	0.612 (0.520–0.721)	<0.001
66–72 years old	0.573 (0.476–0.690)	<0.001	0.452 (0.417–0.490)	<0.001	0.788 (0.653–0.952)	0.013
>72 years old	0.527 (0.449–0.620)	<0.001	0.463 (0.428–0.500)	<0.001	0.878 (0.742–1.038)	0.128
Tumor size						
≤1 cm	0.488 (0.322–0.741)	0.007	0.335 (0.215–0.524)	<0.001	0.686 (0.428–1.100)	0.118
>1 cm, ≤2 cm	0.551 (0.461–0.660)	<0.001	0.396 (0.349–0.450)	<0.001	0.719 (0.598–0.864)	<0.001
>2 cm, ≤3 cm	0.559 (0.467–0.669)	<0.001	0.406 (0.368–0.447)	<0.001	0.726 (0.605–0.872)	<0.001
>3 cm	0.629 (0.528–0.749)	<0.001	0.454 (0.430–0.480)	<0.001	0.722 (0.604–0.863)	<0.001
Radiotherapy or chemotherapy						
No	0.538 (0.451–0.643)	<0.001	0.416 (0.380–0.455)	<0.001	0.773 (0.644–0.927)	0.006
Radiotherapy	0.558 (0.377–0.825)	0.004	0.513 (0.424–0.620)	<0.001	0.919 (0.603–1.402)	0.696
Chemotherapy	0.456 (0.370–0.562)	<0.001	0.356 (0.323–0.391)	<0.001	0.780 (0.633–0.960)	0.019
Both	0.771 (0.664–0.895)	<0.001	0.525 (0.493–0.559)	<0.001	0.681 (0.583–0.795)	<0.001

HRs of multivariable analysis of subgroups were adjusted by year of diagnosis, age, gender, race, marital status, laterality, primary site, histologic type, differentiation, T, tumor size and radiotherapy or chemotherapy except for the subgroup variable itself. NSCLC, non-small cell lung cancer; IPTW, inverse probability of treatment weighting; HR, hazard ratio; CI, confidence interval.

**Table 5:** Multivariable Cox regression analysis of subgroups of all IIIAN2 stage NSCLC patients after IPTW.

Subgroups	Sublobectomy vs. no surgery		Lobectomy vs. no surgery		Lobectomy vs. sublobectomy	
	HR (95%CI)	P	HR (95%CI)	P	HR (95%CI)	P
Age						
<65 years old	0.628 (0.603–0.653)	<0.001	0.391 (0.376–0.407)	<0.001	0.623 (0.597–0.651)	<0.001
66–72 years old	0.660 (0.630–0.691)	<0.001	0.478 (0.458–0.499)	<0.001	0.724 (0.690–0.760)	<0.001
>72 years old	0.643 (0.619–0.668)	<0.001	0.473 (0.455–0.491)	<0.001	0.735 (0.705–0.765)	<0.001
Tumor size						
≤1 cm	0.649 (0.533–0.792)	<0.001	0.414 (0.322–0.533)	<0.001	0.638 (0.504–0.808)	<0.001
>1 cm, ≤2 cm	0.571 (0.536–0.609)	<0.001	0.400 (0.374–0.428)	<0.001	0.700 (0.652–0.752)	<0.001
>2 cm, ≤3 cm	0.545 (0.517–0.575)	<0.001	0.403 (0.381–0.425)	<0.001	0.739 (0.697–0.782)	<0.001
>3 cm	0.661 (0.642–0.680)	<0.001	0.458 (0.446–0.471)	<0.001	0.694 (0.673–0.715)	<0.001
Radiotherapy or chemotherapy						
No	0.525 (0.499–0.553)	<0.001	0.397 (0.378–0.418)	<0.001	0.756 (0.716–0.798)	<0.001
Radiotherapy	0.852 (0.787–0.923)	<0.001	0.512 (0.475–0.552)	<0.001	0.601 (0.549–0.658)	<0.001
Chemotherapy	0.434 (0.411–0.459)	<0.001	0.333 (0.314–0.352)	<0.001	0.766 (0.721–0.814)	<0.001
Both	0.788 (0.762–0.814)	<0.001	0.499 (0.483–0.515)	<0.001	0.633 (0.610–0.656)	<0.001

HRs of multivariable analysis of subgroups were adjusted by year of diagnosis, age, gender, race, marital status, laterality, primary site, histologic type, differentiation, T, tumor size and radiotherapy or chemotherapy except for the subgroup variable itself. NSCLC, non-small cell lung cancer; IPTW, inverse probability of treatment weighting; HR, hazard ratio; CI, confidence interval.

## Discussion

The classic debate regarding whether stage IIIA/N2 NSCLC patients can benefit from lobectomy has gone on for a while with no strong evidence for recommendation, what’s more, no study paid attention to sublobectomy for this population. Some previous randomized controlled trials and retrospective studies showed surgery provided equivalent survival compared with no surgery in stage IIIA/N2 NSCLC patients who underwent chemotherapy or chemoradiotherapy (8–11, 25), while other retrospective studies reported surgery was related with better survival (12–17). Notably, most of these studies included patients underwent pneumonectomy and had small sample size which limited the level of evidence. To explore the influence of sublobectomy and lobectomy on stage IIIA/N2 patients, we performed this multicenter study with a large study cohort using patient information from the SEER database, which captures approximately 28% of the US population (26). In the whole study cohort, we found although there’re much less patients underwent surgery than no surgery patients, both sublobectomy and lobectomy were associated with improved OS rates, and the subgroup analyses based on age, tumor size and radiotherapy or chemotherapy showed similar results. Behera and colleagues’ multicenter retrospective study based on the National Cancer Database revealed that chemoradiotherapy plus lobectomy or pneumonectomy were associated with better survival than only chemoradiotherapy [HR: 0.59 (95%CI: 0.55–0.62), P-value <0.001], which was similar to the result of our subgroup analysis in patients underwent chemoradiotherapy [**Tables 4**, **5**, HR for lobectomy vs. no surgery: 0.525 (95%CI: 0.493–0.559), P-value <0.001; IPTW-adjusted HR for lobectomy vs. no surgery: 0.499 (95%95CI: 0.483–0.515), P-value <0.001] (27). However, like all other studies, this one also didn’t included patients underwent sublobectomy. It’s the first time that our study demonstrated sublobectomy conferred survival benefit compared with no surgery group, and results of subgroup analyses were consistent (**Tables 4**, **5**). Although lobectomy provided better survival prognosis than sublobectomy in all surgery cohort, sublobectomy can still be considered in stage IIIA/N2 NSCLC patients with older age or compromised pulmonary function. However, future randomized controlled trials are needed to further confirm the benefit of lobectomy and sublobectomy for these patients.

For patients confirmed with N2 disease, upfront surgery is not recommended (27). The Radiation Therapy Oncology Group (RTOG) 8808 study compared single radiotherapy with chemotherapy plus radiotherapy in IIIA NSCLC patients and demonstrated a significant better median survival in chemotherapy plus radiotherapy, thus establishing the central role of chemotherapy in the multimodality therapy (28). Concurrent chemoradiotherapy is the standard of care for patients with bulky and/or multi-stational N2 involvement, while for patients with microscopic or minimal lymph node involvement, neoadjuvant chemotherapy plus surgery can confer good survival outcome (11, 27, 29), and Zheng et al. even revealed survival benefit in upfront surgery for some patients with pathological single-station N2 disease (30). The defect of chemoradiotherapy lies in the high rate of local recurrence ranging from 20 to 50% (31). Caglar and colleagues’ study found the rates of local recurrence for IIIA-B patients who received chemoradiotherapy or chemoradiotherapy plus surgery were 50 and 7% (12). Darling et al. also revealed better median survival (50.4 months vs. 20.4 months) and lower regional recurrence (33.7% vs. 51.4%) for induction chemoradiotherapy plus surgery comparing with definitive chemoradiotherapy (17). Although surgery is recommended by many researchers, the rate of surgery for IIIA/N2 patients is low. As shown in **Table 1**, 35.7% of our study cohort received surgery, indicating only a minority of stage IIIA/N2 NSCLC patients have the chance to have the local disease resected. A multidisciplinary panel including a medical oncologist, thoracic surgeon and radiation oncologist should estimate whether the tumor is resectable or unresectable at first (2). The decision of sublobectomy or lobectomy should be made for selected patients cautiously. The high heterogeneity of stage IIIA/N2 NSCLC is owing to differences in primary tumor size and the extent and location of nodal involvement. Complete dissection requires the radical removal of the primary tumor and systemic lymph node sampling and resection. The number of regional nodes examined is a pivotal point for thorough lymph node examination (32, 33). Previous studies focused on resectable early-stage NSCLC recommended number of regional nodes examined should be no less than 11 (34, 35). Our study also showed regional nodes examined ≥12 [**Table S2**, HR: 0.784 (95%CI: 0.720–0.855), P-value <0.001; **Table S3**, IPTW-adjusted HR: 0.780 (95%CI: 0.745–0.817), P-value <0.001] was a protective factor for surgery cohort. As a matter of fact, the percentage of patients with number of regional nodes examined ≥12 is closely related to surgery type as shown in our study (**Table 1**, 8.8% for sublobectomy and 37.0% for lobectomy). Under normal circumstances, thorough intralobar and hilar node examination are technically difficult for sublobectomy. Nevertheless, if the technique of radiological or surgical lymph node evaluation is enhanced, it is promising to combine sublobectomy with thorough lymph node examination or dissection. The stage IIIA/N2 NSCLC patients with reduced lung function can incontrovertibly benefit from these techniques. What’s more, the development in target therapy and immunotherapy will make more stage IIIA/N2 NSCLC resectable.

Another interesting finding in our study was that for patients received surgery, single radiotherapy couldn’t benefit them (**Tables S2**-**S5**, all multivariable Cox regression analysis showed HRs for radiotherapy vs. no radiotherapy or chemotherapy were close to 1). Pless and colleagues conducted a randomized trial comparing induction chemoradiotherapy with induction chemotherapy plus surgery and found radiotherapy did not confer benefit, suggesting one definitive local treatment modality combing neoadjuvant chemotherapy is appropriate to for resectable stage IIIA/N2 NSCLC (36). A subgroup analysis in a multicenter retrospective study performed by Gao et al. also suggested no significant difference in survival outcome between surgery plus chemoradiotherapy and surgery plus chemotherapy, while another subgroup analysis in this research indicated surgery plus radiotherapy exhibited better survival than surgery alone, however, these analyses were univariable based on survival curves (1). Whether radiotherapy can provide benefit for resectable stage IIIA/N2 NSCLC patients especially for these who can’t accept chemotherapy is still unclear. These results should be interpreted with caution and subgroup analyses based on different timing and dose of radiotherapy are needed in the future.

Our research is a population-based multicenter study which reflects the real life, and as far as we know, it is the first study to reveal the benefit sublobectomy confers to stage IIIA/N2 NSCLC patients and compare the survival prognosis of sublobectomy with lobectomy. This research can provide reference in clinical decision and help in future randomized clinical trial design. There are several limitations in our research. First, our research was retrospective and biases were inevitable. Second, the sequence between chemotherapy and surgery or radiotherapy was not recorded in SEER database, as is the case with the detailed information of chemotherapy or radiotherapy. Third, the SEER database didn’t include some confounders like smoking status, respiratory function data, laboratory test results, imaging material, surgery approach (video-assisted thoracic surgery or open surgery), targeted therapy or immunotherapy data which may influence survival outcome and variables of disease-free interval or local recurrence of tumor were also not recorded. Prospective randomized controlled trials are required to further investigate the role of sublobectomy and lobectomy play in stage IIIA/N2 NSCLC.

In conclusion, our study demonstrated that stage IIIA/N2 NSCLC patients could benefit from sublobectomy or lobectomy. Lobectomy provided better OS rate than sublobectomy. However, surgery should be performed in highly selected patients based on the evaluation of a multidisciplinary team.

## Data Availability Statement

The datasets presented in this study can be found in online repositories. The names of the repository/repositories and accession number(s) can be found below: The dataset supporting the conclusions of this article is available in the SEER*Stat software (version 8.3.9; https://seer.cancer.gov/resources/).

## Ethics Statement

Ethical review and approval was not required for the study on human participants in accordance with the local legislation and institutional requirements. Written informed consent from the participants’ legal guardian/next of kin was not required to participate in this study in accordance with the national legislation and the institutional requirements.

## Author Contributions

Conception/design: SW, XS, and ML. Collection and/or assembly of data: XL and ML. Data analysis and interpretation: SW, ZZ, and YG. Manuscript writing: SW, XS, and ML. Final approval of manuscript: all authors. Funding support: XL. All authors contributed to the article and approved the submitted version.

## Funding

This study was funded by the Shanghai “Rising Stars of Medical Talent” Youth Development Program: Outstanding Youth Medical Talents, and Development Fund for the Department of Anesthesiology of Shanghai Pulmonary Hospital.

## Conflict of Interest

The authors declare that the research was conducted in the absence of any commercial or financial relationships that could be construed as a potential conflict of interest.

## Publisher’s Note

All claims expressed in this article are solely those of the authors and do not necessarily represent those of their affiliated organizations, or those of the publisher, the editors and the reviewers. Any product that may be evaluated in this article, or claim that may be made by its manufacturer, is not guaranteed or endorsed by the publisher.

## Abbreviations

AJCC, American Joint Committee on Cancer; CI, confidence interval; HR, hazard ratio; ICD-O-3, International Classification of Diseases for Oncology; IPTW, inverse probability of treatment weighting; IQR, interquartile range; NCCN, National Comprehensive Cancer Network; NOS, not otherwise specified; SEER, Surveillance, Epidemiology, and End Results.
