# Supplementary material for: Lobectomy Versus Sublobectomy in Stage IIIA/N2 Non-Small Cell Lung Cancer: A Population-Based Study
Source: Front Oncol. 2021 Dec 9;11:726811. doi: 10.3389/fonc.2021.726811 (PMC8696201; doi:10.3389/fonc.2021.726811)
Supplement: Supplementary file 1 [file DataSheet_1.docx]

Supplementary Material

**TABLE S1** Selection procedure of study cohort

| Step | Criteria | Number excluded | Number remained |
| --- | --- | --- | --- |
| 1 | Patients with T1-3N2M0 NSCLC between 2004 and 2015 | - | 38,639 |
| 2 | Include if identified as only one primary tumor | 6,109 | 26,872 |
| 3 | Include if aged ≥18 years | 3 | 26,869 |
| 4 | Include if treated with no surgery, sublobectomy or lobectomy | 1,109 | 25,760 |
| 5 | Exclude if diagnosed with autopsy/death certificate only or diagnosed without pathologically confirmed | 217 | 25,543 |
| 6 | Exclude if survival time of less than 1 month after diagnosis | 1,002 | 24,541 |
| 7 | Exclude if missing information about variables required by our study: 110 with unknown specific surgery strategy, 38 with unknown race, 45 with unknown laterality, 1671 with unknown tumor size, 775 with unknown marital status at diagnosis, 264 unknown if radiotherapy performed. | 2,903 | 21,638 |

NSCLC, non-small cell lung cancer.

**TABLE S2** Cox regression analysis of HR for regional nodes examined in IIIAN2 stage NSCLC patients underwent sublobectomy or lobectomy before IPTW

| Variables | Univariable analysis | |  | Multivariable analysis | |
| --- | --- | --- | --- | --- | --- |
|  | HR (95%CI) | P |  | HR (95%CI) | P |
| Regional nodes examined |  |  |  |  |  |
| ≤5 | 1 |  |  | 1 |  |
| 6-11 | 0.851 (0.782-0.926) | <0.001 |  | 0.877 (0.805-0.955) | 0.003 |
| ≥12 | 0.783 (0.719-0.852) | <0.001 |  | 0.784 (0.720-0.855) | <0.001 |
| Unknown | 0.832 (0.740-0.936) | 0.002 |  | 0.837 (0.743-0.942) | 0.003 |
| Year of diagnosis |  |  |  |  |  |
| 2004-2009 | 1 |  |  | 1 |  |
| 2010-2015 | 0.806 (0.752-0.863) | <0.001 |  | 0.815 (0.759-0.874) | <0.001 |
| Age |  |  |  |  |  |
| <65 years old | 1 |  |  | 1 |  |
| 66-72 years old | 1.388 (1.284-1.500) | <0.001 |  | 1.354 (1.251-1.466) | <0.001 |
| >72 years old | 1.823 (1.685-1.972) | <0.001 |  | 1.705 (1.567-1.855) | <0.001 |
| Gender |  |  |  |  |  |
| Male | 1 |  |  | 1 |  |
| Female | 0.750 (0.703-0.800) | <0.001 |  | 0.746 (0.697-0.799) | <0.001 |
| Race |  |  |  |  |  |
| White | 1 |  |  | 1 |  |
| Black | 0.906 (0.810-1.015) | 0.088 |  | 0.966 (0.861-1.085) | 0.559 |
| Other | 0.837 (0.742-0.943) | 0.004 |  | 0.841 (0.745-0.949) | 0.005 |
| Marital status |  |  |  |  |  |
| Married | 1 |  |  | 1 |  |
| Unmarried | 0.984 (0.883-1.097) | 0.775 |  | 1.115 (0.998-1.247) | 0.055 |
| Seperated/Divorced/Widowed | 1.143 (1.062-1.230) | <0.001 |  | 1.121 (1.037-1.212) | 0.004 |
| Laterality |  |  |  |  |  |
| Right | 1 |  |  |  |  |
| Left | 1.032 (0.967-1.102) | 0.344 |  |  |  |
| Primary site |  |  |  |  |  |
| Main bronchus | 1 |  |  |  |  |
| Upper lobe | 0.806 (0.433-1.499) | 0.495 |  |  |  |
| Middle lobe | 0.756 (0.400-1.430) | 0.390 |  |  |  |
| Lower lobe | 0.928 (0.498-1.729) | 0.814 |  |  |  |
| Overlapping lesion of lung | 1.048 (0.534-2.057) | 0.890 |  |  |  |
| Unknown | 1.051 (0.514-2.149) | 0.893 |  |  |  |
| Histologic type |  |  |  |  |  |
| Adenocarcinoma | 1 |  |  | 1 |  |
| Squamous cell | 1.233 (1.137-1.338) | <0.001 |  | 1.039 (0.955-1.131) | 0.374 |
| Other | 1.018 (0.929-1.116) | 0.698 |  | 0.961 (0.870-1.060) | 0.424 |
| Differentiation |  |  |  |  |  |
| Grade I | 1 |  |  | 1 |  |
| Grade II | 1.368 (1.160-1.615) | <0.001 |  | 1.371 (1.160-1.620) | <0.001 |
| Grade III | 1.552 (1.318-1.829) | <0.001 |  | 1.542 (1.307-1.819) | <0.001 |
| Grade IV | 1.712 (1.323-2.215) | <0.001 |  | 1.596 (1.226-2.079) | <0.001 |
| Unknown | 1.106 (0.916-1.336) | 0.293 |  | 1.129 (0.932-1.367) | 0.215 |
| T |  |  |  |  |  |
| T1 | 1 |  |  | 1 |  |
| T2 | 1.307 (1.210-1.412) | <0.001 |  | 1.177 (1.064-1.303) | 0.002 |
| T3 | 1.591 (1.448-1.748) | <0.001 |  | 1.490 (1.329-1.670) | <0.001 |
| Tumor size |  |  |  |  |  |
| ≤1cm | 1 |  |  | 1 |  |
| >1cm, ≤2cm | 1.031 (0.814-1.306) | 0.798 |  | 0.979 (0.772-1.240) | 0.858 |
| >2cm, ≤3cm | 1.245 (0.987-1.571) | 0.064 |  | 1.152 (0.911-1.456) | 0.237 |
| >3cm | 1.453 (1.157-1.824) | 0.001 |  | 1.197 (0.941-1.523) | 0.143 |
| Radiotherapy or chemotherapy |  |  |  |  |  |
| No | 1 |  |  | 1 |  |
| Radiotherapy | 1.030 (0.869-1.221) | 0.736 |  | 1.007 (0.849-1.195) | 0.933 |
| Chemotherapy | 0.683 (0.626-0.745) | <0.001 |  | 0.745 (0.681-0.815) | <0.001 |
| Both | 0.696 (0.642-0.754) | <0.001 |  | 0.783 (0.719-0.853) | <0.001 |

HR, hazard ratio; NSCLC, non-small cell lung cancer; IPTW, inverse probability of treatment weighting; CI, confidence interval.

**TABLE S3** Cox regression analysis of HR for regional nodes examined in IIIAN2 stage NSCLC patients underwent sublobectomy or lobectomy after IPTW

| Variables | Univariable analysis | |  | Multivariable analysis | |
| --- | --- | --- | --- | --- | --- |
|  | HR (95%CI) | P |  | HR (95%CI) | P |
| Regional nodes examined |  |  |  |  |  |
| ≤5 | 1 |  |  | 1 |  |
| 6-11 | 0.858 (0.820-0.898) | <0.001 |  | 0.873 (0.834-0.914) | <0.001 |
| ≥12 | 0.787 (0.752-0.824) | <0.001 |  | 0.780 (0.745-0.817) | <0.001 |
| Unknown | 0.836 (0.798-0.876) | <0.001 |  | 0.833 (0.795-0.872) | <0.001 |
| Year of diagnosis |  |  |  |  |  |
| 2004-2009 | 1 |  |  | 1 |  |
| 2010-2015 | 0.834 (0.805-0.864) | <0.001 |  | 0.827 (0.798-0.857) | <0.001 |
| Age |  |  |  |  |  |
| <65 years old | 1 |  |  | 1 |  |
| 66-72 years old | 1.344 (1.291-1.399) | <0.001 |  | 1.295 (1.243-1.349) | <0.001 |
| >72 years old | 1.807 (1.735-1.881) | <0.001 |  | 1.684 (1.613-1.758) | <0.001 |
| Gender |  |  |  |  |  |
| Male | 1 |  |  | 1 |  |
| Female | 0.745 (0.720-0.770) | <0.001 |  | 0.744 (0.718-0.770) | <0.001 |
| Race |  |  |  |  |  |
| White | 1 |  |  | 1 |  |
| Black | 0.887 (0.836-0.942) | <0.001 |  | 0.950 (0.894-1.010) | 0.103 |
| Other | 0.812 (0.763-0.865) | <0.001 |  | 0.836 (0.785-0.891) | <0.001 |
| Marital status |  |  |  |  |  |
| Married | 1 |  |  | 1 |  |
| Unmarried | 0.985 (0.931-1.041) | 0.589 |  | 1.122 (1.059-1.188) | <0.001 |
| Seperated/Divorced/Widowed | 1.135 (1.093-1.178) | <0.001 |  | 1.131 (1.087-1.177) | <0.001 |
| Laterality |  |  |  |  |  |
| Right | 1 |  |  | 1 |  |
| Left | 1.030 (0.996-1.065) | 0.086 |  | 1.029 (0.994-1.064) | 0.102 |
| Primary site |  |  |  |  |  |
| Main bronchus | 1 |  |  |  |  |
| Upper lobe | 0.756 (0.497-1.150) | 0.191 |  |  |  |
| Middle lobe | 0.751 (0.490-1.150) | 0.187 |  |  |  |
| Lower lobe | 0.865 (0.569-1.317) | 0.500 |  |  |  |
| Overlapping lesion of lung | 0.865 (0.554-1.351) | 0.524 |  |  |  |
| Unknown | 1.070 (0.668-1.713) | 0.778 |  |  |  |
| Histologic type |  |  |  |  |  |
| Adenocarcinoma | 1 |  |  | 1 |  |
| Squamous cell | 1.257 (1.206-1.310) | <0.001 |  | 1.051 (1.006-1.098) | 0.025 |
| Other | 1.014 (0.967-1.064) | 0.560 |  | 0.969 (0.921-1.021) | 0.238 |
| Differentiation |  |  |  |  |  |
| Grade I | 1 |  |  | 1 |  |
| Grade II | 1.425 (1.306-1.554) | <0.001 |  | 1.430 (1.310-1.561) | <0.001 |
| Grade III | 1.663 (1.526-1.813) | <0.001 |  | 1.651 (1.514-1.801) | <0.001 |
| Grade IV | 1.799 (1.573-2.058) | <0.001 |  | 1.726 (1.503-1.981) | <0.001 |
| Unknown | 1.124 (1.018-1.241) | 0.021 |  | 1.166 (1.054-1.289) | 0.003 |
| T |  |  |  |  |  |
| T1 | 1 |  |  | 1 |  |
| T2 | 1.309 (1.259-1.362) | <0.001 |  | 1.162 (1.103-1.225) | <0.001 |
| T3 | 1.540 (1.468-1.616) | <0.001 |  | 1.417 (1.337-1.503) | <0.001 |
| Tumor size |  |  |  |  |  |
| ≤1cm | 1 |  |  | 1 |  |
| >1cm, ≤2cm | 1.041 (0.917-1.181) | 0.538 |  | 0.983 (0.866-1.116) | 0.787 |
| >2cm, ≤3cm | 1.243 (1.098-1.408) | <0.001 |  | 1.135 (1.002-1.287) | 0.047 |
| >3cm | 1.470 (1.301-1.661) | <0.001 |  | 1.180 (1.038-1.341) | 0.011 |
| Radiotherapy or chemotherapy |  |  |  |  |  |
| No | 1 |  |  | 1 |  |
| Radiotherapy | 0.982 (0.895-1.079) | 0.712 |  | 0.934 (0.850-1.027) | 0.159 |
| Chemotherapy | 0.690 (0.660-0.722) | <0.001 |  | 0.743 (0.710-0.778) | <0.001 |
| Both | 0.687 (0.659-0.716) | <0.001 |  | 0.769 (0.736-0.803) | <0.001 |

HR, hazard ratio; NSCLC, non-small cell lung cancer; IPTW, inverse probability of treatment weighting; CI, confidence interval.

**TABLE S4** Cox regression analysis of HR for regional nodes positive in IIIAN2 stage NSCLC patients underwent sublobectomy or lobectomy before IPTW

| Variables | Univariable analysis | |  | Multivariable analysis | |
| --- | --- | --- | --- | --- | --- |
|  | HR (95%CI) | P |  | HR (95%CI) | P |
| Regional nodes positive |  |  |  |  |  |
| ≤5 | 1 |  |  | 1 |  |
| 6-11 | 1.186 (1.087-1.294) | <0.001 |  | 1.193 (1.093-1.304) | <0.001 |
| ≥12 | 1.471 (1.353-1.600) | <0.001 |  | 1.509 (1.386-1.643) | <0.001 |
| Unknown | 1.251 (1.126-1.389) | <0.001 |  | 1.267 (1.139-1.409) | <0.001 |
| Year of diagnosis |  |  |  |  |  |
| 2004-2009 | 1 |  |  | 1 |  |
| 2010-2015 | 0.806 (0.752-0.863) | <0.001 |  | 0.789 (0.736-0.846) | <0.001 |
| Age |  |  |  |  |  |
| <65 years old | 1 |  |  | 1 |  |
| 66-72 years old | 1.388 (1.284-1.500) | <0.001 |  | 1.354 (1.251-1.465) | <0.001 |
| >72 years old | 1.823 (1.685-1.972) | <0.001 |  | 1.699 (1.562-1.848) | <0.001 |
| Gender |  |  |  |  |  |
| Male | 1 |  |  | 1 |  |
| Female | 0.750 (0.703-0.800) | <0.001 |  | 0.749 (0.699-0.802) | <0.001 |
| Race |  |  |  |  |  |
| White | 1 |  |  | 1 |  |
| Black | 0.906 (0.810-1.015) | 0.088 |  | 0.980 (0.873-1.100) | 0.734 |
| Other | 0.837 (0.742-0.943) | 0.004 |  | 0.857 (0.759-0.967) | 0.012 |
| Marital status |  |  |  |  |  |
| Married | 1 |  |  | 1 |  |
| Unmarried | 0.984 (0.883-1.097) | 0.775 |  | 1.121 (1.003-1.253) | 0.045 |
| Seperated/Divorced/Widowed | 1.143 (1.062-1.230) | 0.000 |  | 1.138 (1.052-1.230) | 0.001 |
| Laterality |  |  |  |  |  |
| Right | 1 |  |  |  |  |
| Left | 1.032 (0.967-1.102) | 0.344 |  |  |  |
| Primary site |  |  |  |  |  |
| Main bronchus | 1 |  |  |  |  |
| Upper lobe | 0.806 (0.433-1.499) | 0.495 |  |  |  |
| Middle lobe | 0.756 (0.400-1.430) | 0.390 |  |  |  |
| Lower lobe | 0.928 (0.498-1.729) | 0.814 |  |  |  |
| Overlapping lesion of lung | 1.048 (0.534-2.057) | 0.890 |  |  |  |
| Unknown | 1.051 (0.514-2.149) | 0.893 |  |  |  |
| Histologic type |  |  |  |  |  |
| Adenocarcinoma | 1 |  |  | 1 |  |
| Squamous cell | 1.233 (1.137-1.338) | <0.001 |  | 1.092 (1.002-1.189) | 0.044 |
| Other | 1.018 (0.929-1.116) | 0.698 |  | 0.978 (0.886-1.080) | 0.660 |
| Differentiation |  |  |  |  |  |
| Grade I | 1 |  |  | 1 |  |
| Grade II | 1.368 (1.160-1.615) | <0.001 |  | 1.345 (1.138-1.589) | <0.001 |
| Grade III | 1.552 (1.318-1.829) | <0.001 |  | 1.526 (1.293-1.800) | <0.001 |
| Grade IV | 1.712 (1.323-2.215) | <0.001 |  | 1.578 (1.212-2.055) | <0.001 |
| Unknown | 1.106 (0.916-1.336) | 0.293 |  | 1.145 (0.945-1.387) | 0.168 |
| T |  |  |  |  |  |
| T1 | 1 |  |  | 1 |  |
| T2 | 1.307 (1.210-1.412) | <0.001 |  | 1.160 (1.048-1.284) | 0.004 |
| T3 | 1.591 (1.448-1.748) | <0.001 |  | 1.461 (1.304-1.638) | <0.001 |
| Tumor size |  |  |  |  |  |
| ≤1cm | 1 |  |  | 1 |  |
| >1cm, ≤2cm | 1.031 (0.814-1.306) | 0.798 |  | 0.933 (0.735-1.183) | 0.565 |
| >2cm, ≤3cm | 1.245 (0.987-1.571) | 0.064 |  | 1.066 (0.843-1.348) | 0.594 |
| >3cm | 1.453 (1.157-1.824) | 0.001 |  | 1.106 (0.869-1.407) | 0.413 |
| Radiotherapy or chemotherapy |  |  |  |  |  |
| No | 1 |  |  | 1 |  |
| Radiotherapy | 1.030 (0.869-1.221) | 0.736 |  | 1.032 (0.870-1.225) | 0.714 |
| Chemotherapy | 0.683 (0.626-0.745) | <0.001 |  | 0.723 (0.661-0.791) | <0.001 |
| Both | 0.696 (0.642-0.754) | <0.001 |  | 0.760 (0.697-0.828) | <0.001 |

HR, hazard ratio; NSCLC, non-small cell lung cancer; IPTW, inverse probability of treatment weighting; CI, confidence interval.

**TABLE S5** Cox regression analysis of HR for regional nodes positive in IIIAN2 stage NSCLC patients underwent sublobectomy or lobectomy after IPTW

| Variables | Univariable analysis | |  | Multivariable analysis | |
| --- | --- | --- | --- | --- | --- |
|  | HR (95%CI) | P |  | HR (95%CI) | P |
| Regional nodes positive |  |  |  |  |  |
| ≤5 | 1 |  |  | 1 |  |
| 6-11 | 1.166 (1.113-1.223) | <0.001 |  | 1.185 (1.130-1.242) | <0.001 |
| ≥12 | 1.440 (1.375-1.508) | <0.001 |  | 1.490 (1.422-1.561) | <0.001 |
| Unknown | 1.245 (1.188-1.305) | <0.001 |  | 1.258 (1.200-1.319) | <0.001 |
| Year of diagnosis |  |  |  |  |  |
| 2004-2009 | 1 |  |  | 1 |  |
| 2010-2015 | 0.788 (0.761-0.816) | <0.001 |  | 0.780 (0.753-0.808) | <0.001 |
| Age |  |  |  |  |  |
| <65 years old | 1 |  |  | 1 |  |
| 66-72 years old | 1.311 (1.260-1.363) | <0.001 |  | 1.271 (1.221-1.324) | <0.001 |
| >72 years old | 1.723 (1.655-1.793) | <0.001 |  | 1.586 (1.519-1.655) | <0.001 |
| Gender |  |  |  |  |  |
| Male | 1 |  |  | 1 |  |
| Female | 0.737 (0.713-0.762) | <0.001 |  | 0.734 (0.709-0.760) | <0.001 |
| Race |  |  |  |  |  |
| White | 1 |  |  | 1 |  |
| Black | 0.871 (0.821-0.924) | <0.001 |  | 0.923 (0.869-0.981) | 0.010 |
| Other | 0.782 (0.734-0.833) | <0.001 |  | 0.827 (0.777-0.881) | <0.001 |
| Marital status |  |  |  |  |  |
| Married | 1 |  |  | 1 |  |
| Unmarried | 0.994 (0.940-1.050) | 0.819 |  | 1.126 (1.063-1.192) | <0.001 |
| Seperated/Divorced/Widowed | 1.203 (1.159-1.248) | <0.001 |  | 1.200 (1.153-1.248) | <0.001 |
| Laterality |  |  |  |  |  |
| Right | 1 |  |  | 1 |  |
| Left | 1.078 (1.043-1.114) | <0.001 |  | 1.086 (1.050-1.122) | <0.001 |
| Primary site |  |  |  |  |  |
| Main bronchus | 1 |  |  |  |  |
| Upper lobe | 0.877 (0.587-1.309) | 0.520 |  |  |  |
| Middle lobe | 0.870 (0.579-1.307) | 0.502 |  |  |  |
| Lower lobe | 0.991 (0.664-1.480) | 0.965 |  |  |  |
| Overlapping lesion of lung | 1.014 (0.662-1.555) | 0.948 |  |  |  |
| Unknown | 1.135 (0.720-1.788) | 0.586 |  |  |  |
| Histologic type |  |  |  |  |  |
| Adenocarcinoma | 1 |  |  | 1 |  |
| Squamous cell | 1.287 (1.235-1.342) | <0.001 |  | 1.082 (1.037-1.130) | <0.001 |
| Other | 1.001 (0.955-1.050) | 0.951 |  | 0.925 (0.879-0.974) | 0.003 |
| Differentiation |  |  |  |  |  |
| Grade I | 1 |  |  | 1 |  |
| Grade II | 1.386 (1.272-1.511) | <0.001 |  | 1.403 (1.286-1.530) | <0.001 |
| Grade III | 1.611 (1.479-1.754) | <0.001 |  | 1.640 (1.505-1.788) | <0.001 |
| Grade IV | 1.628 (1.413-1.875) | <0.001 |  | 1.662 (1.437-1.921) | <0.001 |
| Unknown | 1.180 (1.070-1.301) | <0.001 |  | 1.277 (1.156-1.411) | <0.001 |
| T |  |  |  |  |  |
| T1 | 1 |  |  | 1 |  |
| T2 | 1.317 (1.266-1.370) | <0.001 |  | 1.162 (1.103-1.224) | <0.001 |
| T3 | 1.594 (1.519-1.674) | <0.001 |  | 1.469 (1.385-1.557) | <0.001 |
| Tumor size |  |  |  |  |  |
| ≤1cm | 1 |  |  | 1 |  |
| >1cm, ≤2cm | 0.915 (0.804-1.041) | 0.178 |  | 0.847 (0.743-0.965) | 0.012 |
| >2cm, ≤3cm | 1.086 (0.955-1.234) | 0.207 |  | 0.973 (0.856-1.107) | 0.682 |
| >3cm | 1.316 (1.161-1.492) | <0.001 |  | 1.038 (0.910-1.185) | 0.575 |
| Radiotherapy or chemotherapy |  |  |  |  |  |
| No | 1 |  |  | 1 |  |
| Radiotherapy | 1.027 (0.936-1.126) | 0.574 |  | 1.053 (0.959-1.156) | 0.276 |
| Chemotherapy | 0.704 (0.673-0.736) | <0.001 |  | 0.755 (0.721-0.791) | <0.001 |
| Both | 0.710 (0.682-0.740) | <0.001 |  | 0.791 (0.757-0.827) | <0.001 |

HR, hazard ratio; NSCLC, non-small cell lung cancer; IPTW, inverse probability of treatment weighting; CI, confidence interval.

**TABLE S6** Cox regression analysis of IIIAN2 stage NSCLC patients who had no surgery or surgery with neoadjuvant therapy

| Variables | Univariable analysis | |  | Multivariable analysis | |
| --- | --- | --- | --- | --- | --- |
|  | HR (95%CI) | P |  | HR (95%CI) | P |
| Surgery |  |  |  |  |  |
| Sublobectomy vs. no surgery | 0.491 (0.351-0.688) | <0.001 |  | 0.607 (0.433-0.851) | 0.004 |
| Lobectomy vs. no surgery | 0.376 (0.347-0.408) | <0.001 |  | 0.464 (0.426-0.504) | <0.001 |
| Lobectomy vs. sublobectomy | 0.765 (0.542-1.081) | 0.129 |  | 0.764 (0.540-1.079) | 0.126 |
| Year of diagnosis |  |  |  |  |  |
| 2004-2009 | 1 |  |  | 1 |  |
| 2010-2015 | 0.881 (0.852-0.910) | <0.001 |  | 0.918 (0.887-0.950) | <0.001 |
| Age |  |  |  |  |  |
| <65 years old | 1 |  |  | 1 |  |
| 66-72 years old | 1.207 (1.156-1.259) | <0.001 |  | 1.141 (1.092-1.192) | <0.001 |
| >72 years old | 1.603 (1.542-1.667) | <0.001 |  | 1.307 (1.254-1.363) | <0.001 |
| Gender |  |  |  |  |  |
| Male | 1 |  |  | 1 |  |
| Female | 0.846 (0.818-0.874) | <0.001 |  | 0.852 (0.823-0.883) | <0.001 |
| Race |  |  |  |  |  |
| White | 1 |  |  | 1 |  |
| Black | 0.915 (0.872-0.961) | <0.001 |  | 0.925 (0.880-0.973) | 0.002 |
| Other | 0.859 (0.801-0.921) | <0.001 |  | 0.849 (0.792-0.911) | <0.001 |
| Marital status |  |  |  |  |  |
| Married | 1 |  |  | 1 |  |
| Unmarried | 0.978 (0.929-1.029) | 0.384 |  | 1.019 (0.967-1.074) | 0.482 |
| Seperated/Divorced/Widowed | 1.153 (1.112-1.195) | <0.001 |  | 1.107 (1.066-1.149) | <0.001 |
| Laterality |  |  |  |  |  |
| Right | 1 |  |  |  |  |
| Left | 1.019 (0.985-1.054) | 0.285 |  |  |  |
| Primary site |  |  |  |  |  |
| Main bronchus | 1 |  |  | 1 |  |
| Upper lobe | 0.902 (0.829-0.982) | 0.017 |  | 0.942 (0.864-1.025) | 0.166 |
| Middle lobe | 0.959 (0.857-1.073) | 0.463 |  | 1.040 (0.929-1.165) | 0.498 |
| Lower lobe | 1.052 (0.964-1.149) | 0.255 |  | 1.060 (0.970-1.158) | 0.198 |
| Overlapping lesion of lung | 0.915 (0.746-1.123) | 0.397 |  | 0.934 (0.761-1.147) | 0.514 |
| Unknown | 1.111 (0.971-1.273) | 0.126 |  | 1.061 (0.926-1.216) | 0.391 |
| Histologic type |  |  |  |  |  |
| Adenocarcinoma | 1 |  |  | 1 |  |
| Squamous cell | 1.360 (1.309-1.412) | <0.001 |  | 1.173 (1.127-1.220) | <0.001 |
| Other | 1.291 (1.237-1.348) | <0.001 |  | 1.144 (1.094-1.197) | <0.001 |
| Differentiation |  |  |  |  |  |
| Grade I | 1 |  |  | 1 |  |
| Grade II | 1.164 (1.043-1.299) | 0.006 |  | 1.187 (1.063-1.326) | 0.002 |
| Grade III | 1.189 (1.070-1.321) | 0.001 |  | 1.230 (1.106-1.369) | <0.001 |
| Grade IV | 1.309 (1.117-1.535) | <0.001 |  | 1.357 (1.155-1.595) | <0.001 |
| Unknown | 1.165 (1.049-1.293) | 0.004 |  | 1.154 (1.038-1.282) | 0.008 |
| T |  |  |  |  |  |
| T1 | 1 |  |  | 1 |  |
| T2 | 1.235 (1.182-1.290) | <0.001 |  | 1.043 (0.974-1.118) | 0.227 |
| T3 | 1.388 (1.325-1.455) | <0.001 |  | 1.219 (1.139-1.305) | <0.001 |
| Tumor size |  |  |  |  |  |
| ≤1cm | 1 |  |  | 1 |  |
| >1cm, ≤2cm | 1.039 (0.897-1.204) | 0.609 |  | 0.978 (0.844-1.134) | 0.768 |
| >2cm, ≤3cm | 1.237 (1.072-1.429) | 0.004 |  | 1.136 (0.983-1.313) | 0.084 |
| >3cm | 1.457 (1.268-1.676) | <0.001 |  | 1.275 (1.100-1.479) | 0.001 |
| Radiotherapy or chemotherapy |  |  |  |  |  |
| No | 1 |  |  | 1 |  |
| Radiotherapy | 0.754 (0.712-0.798) | <0.001 |  | 0.716 (0.676-0.758) | <0.001 |
| Chemotherapy | 0.549 (0.518-0.582) | <0.001 |  | 0.637 (0.600-0.676) | <0.001 |
| Both | 0.422 (0.404-0.441) | <0.001 |  | 0.461 (0.441-0.482) | <0.001 |

NSCLC, non-small cell lung cancer; HR, hazard ratio; CI, confidence interval.

**TABLE S7** Cox regression analysis of IIIAN2 stage NSCLC patients who had no surgery or surgery without neoadjuvant therapy

| Variables | Univariable analysis | |  | Multivariable analysis | |
| --- | --- | --- | --- | --- | --- |
|  | HR (95%CI) | P |  | HR (95%CI) | P |
| Surgery |  |  |  |  |  |
| Sublobectomy vs. no surgery | 0.572 (0.519-0.630) | <0.001 |  | 0.585 (0.529-0.647) | <0.001 |
| Lobectomy vs. no surgery | 0.449 (0.430-0.468) | <0.001 |  | 0.437 (0.417-0.459) | <0.001 |
| Lobectomy vs. sublobectomy | 0.785 (0.709-0.870) | <0.001 |  | 0.747 (0.674-0.829) | <0.001 |
| Year of diagnosis |  |  |  |  |  |
| 2004-2009 | 1 |  |  | 1 |  |
| 2010-2015 | 0.893 (0.866-0.921) | <0.001 |  | 0.900 (0.872-0.929) | <0.001 |
| Age |  |  |  |  |  |
| <65 years old | 1 |  |  | 1 |  |
| 66-72 years old | 1.245 (1.197-1.295) | <0.001 |  | 1.172 (1.126-1.220) | <0.001 |
| >72 years old | 1.645 (1.586-1.705) | <0.001 |  | 1.347 (1.296-1.400) | <0.001 |
| Gender |  |  |  |  |  |
| Male | 1 |  |  | 1 |  |
| Female | 0.807 (0.783-0.832) | <0.001 |  | 0.832 (0.806-0.860) | <0.001 |
| Race |  |  |  |  |  |
| White | 1 |  |  | 1 |  |
| Black | 0.939 (0.897-0.983) | 0.007 |  | 0.927 (0.884-0.972) | 0.002 |
| Other | 0.814 (0.765-0.867) | <0.001 |  | 0.850 (0.798-0.906) | <0.001 |
| Marital status |  |  |  |  |  |
| Married | 1 |  |  | 1 |  |
| Unmarried | 1.001 (0.954-1.050) | 0.965 |  | 1.029 (0.980-1.082) | 0.25 |
| Seperated/Divorced/Widowed | 1.179 (1.141-1.219) | <0.001 |  | 1.111 (1.073-1.151) | <0.001 |
| Laterality |  |  |  |  |  |
| Right | 1 |  |  | 1 |  |
| Left | 0.952 (0.922-0.982) | 0.002 |  | 0.997 (0.965-1.029) | 0.831 |
| Primary site |  |  |  |  |  |
| Main bronchus | 1 |  |  | 1 |  |
| Upper lobe | 0.821 (0.755-0.893) | <0.001 |  | 0.956 (0.878-1.041) | 0.301 |
| Middle lobe | 0.812 (0.729-0.906) | <0.001 |  | 1.012 (0.906-1.130) | 0.831 |
| Lower lobe | 0.880 (0.807-0.959) | 0.004 |  | 1.058 (0.969-1.155) | 0.206 |
| Overlapping lesion of lung | 0.880 (0.734-1.055) | 0.167 |  | 1.037 (0.864-1.244) | 0.697 |
| Unknown | 1.088 (0.953-1.242) | 0.214 |  | 1.089 (0.953-1.244) | 0.209 |
| Histologic type |  |  |  |  |  |
| Adenocarcinoma | 1 |  |  | 1 |  |
| Squamous cell | 1.496 (1.445-1.549) | <0.001 |  | 1.151 (1.109-1.194) | <0.001 |
| Other | 1.360 (1.308-1.415) | <0.001 |  | 1.101 (1.056-1.148) | <0.001 |
| Differentiation |  |  |  |  |  |
| Grade I | 1 |  |  | 1 |  |
| Grade II | 1.150 (1.046-1.264) | 0.004 |  | 1.228 (1.117-1.351) | <0.001 |
| Grade III | 1.384 (1.262-1.517) | <0.001 |  | 1.312 (1.195-1.440) | <0.001 |
| Grade IV | 1.541 (1.339-1.773) | <0.001 |  | 1.467 (1.272-1.691) | <0.001 |
| Unknown | 1.505 (1.374-1.649) | <0.001 |  | 1.206 (1.099-1.324) | <0.001 |
| T |  |  |  |  |  |
| T1 | 1 |  |  | 1 |  |
| T2 | 1.304 (1.254-1.357) | <0.001 |  | 1.087 (1.025-1.152) | 0.006 |
| T3 | 1.605 (1.537-1.675) | <0.001 |  | 1.283 (1.209-1.362) | <0.001 |
| Tumor size |  |  |  |  |  |
| ≤1cm | 1 |  |  | 1 |  |
| >1cm, ≤2cm | 0.992 (0.870-1.132) | 0.909 |  | 1.000 (0.876-1.141) | 0.998 |
| >2cm, ≤3cm | 1.219 (1.072-1.387) | 0.003 |  | 1.169 (1.027-1.332) | 0.018 |
| >3cm | 1.598 (1.410-1.811) | <0.001 |  | 1.288 (1.128-1.471) | <0.001 |
| Radiotherapy or chemotherapy |  |  |  |  |  |
| No | 1 |  |  | 1 |  |
| Radiotherapy | 1.057 (1.003-1.115) | 0.039 |  | 0.768 (0.728-0.811) | <0.001 |
| Chemotherapy | 0.610 (0.580-0.641) | <0.001 |  | 0.669 (0.636-0.703) | <0.001 |
| Both | 0.615 (0.591-0.639) | <0.001 |  | 0.504 (0.484-0.525) | <0.001 |

NSCLC, non-small cell lung cancer; HR, hazard ratio; CI, confidence interval.

**FIGURE CAPTION**

**Figure S1** Absolute standard difference of coviariates (year of diagnosis, age, gender, race, marital status, laterality, primary site, histologic type, differentiation, T, tumor size, radiotherapy or chemotherapy) for analysis of surgical strategy (A), regional nodes examined (B), and regional nodes positive (C) before and after IPTW. Blue lines means a reduction, while red lines means an increase in absolute standard difference. Closed red circles indicate a statistically signicant difference and hollow red circles indicate a not statistically significant difference. IPTW, inverse probability of treatment weighting.
