# Supplementary figures and images for: Lobectomy Versus Sublobectomy in Stage IIIA/N2 Non-Small Cell Lung Cancer: A Population-Based Study
Source: Front Oncol. 2021 Dec 9;11:726811. doi: 10.3389/fonc.2021.726811 (PMC8696201; doi:10.3389/fonc.2021.726811)

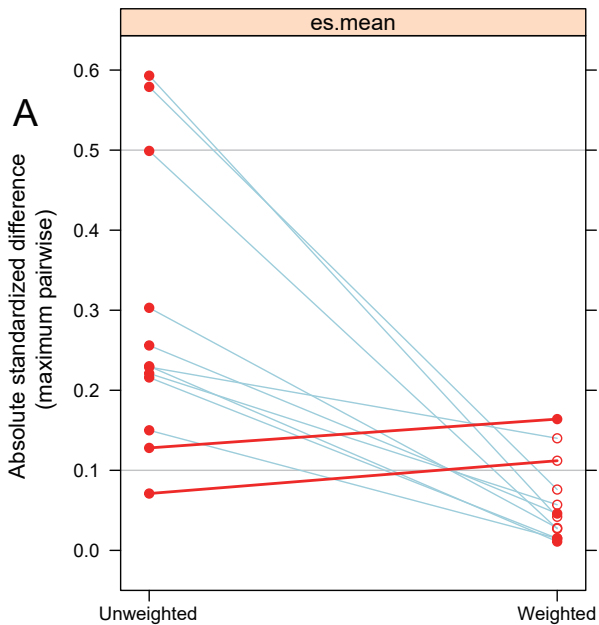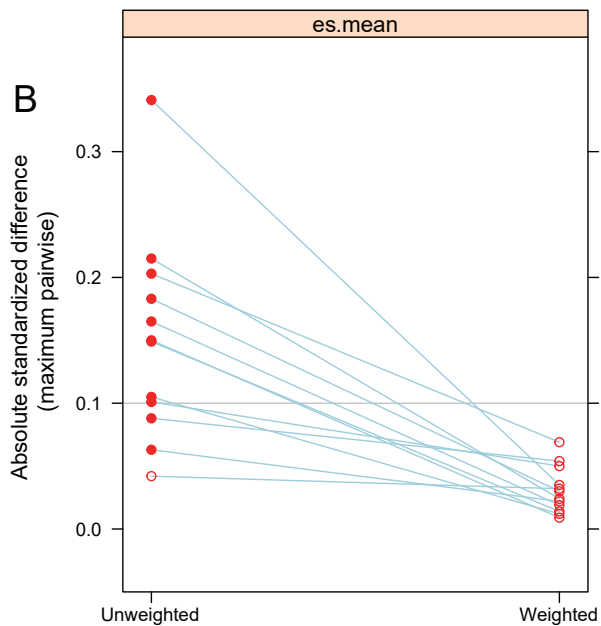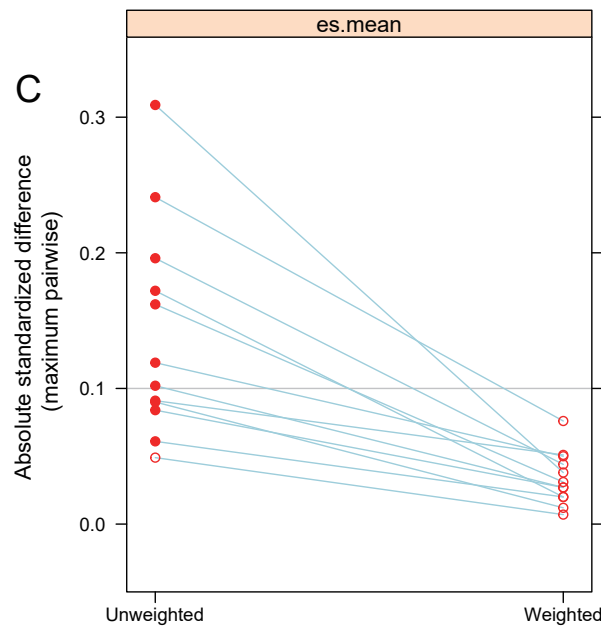

Supplement: Supplementary file 2 [file Image_1.pdf]
